# Effects of Different Stages of Training on the Intestinal Microbes of Yili Horses Analyzed Using Metagenomics

**DOI:** 10.3390/genes16050504

**Published:** 2025-04-27

**Authors:** Yuan-Fang Sun, Zi-Xiang Han, Xin-Kui Yao, Jun Meng, Wan-Lu Ren, Chuan-Kun Wang, Xin-Xin Yuan, Ya-Qi Zeng, Yong-Fa Wang, Zhi-Wen Sun, Jian-Wen Wang

**Affiliations:** 1 College of Animal Science, Xinjiang Agricultural University, Urumqi 830052, China; 15809926110@163.com (Y.-F.S.); hzx15688350629@163.com (Z.-X.H.); yxk61@126.com (X.-K.Y.); junm86@sina.com (J.M.); 13201295117@163.com (W.-L.R.); wck94214@163.com (C.-K.W.); xjauyxx@126.com (X.-X.Y.); xjauzengyaqi@163.com (Y.-Q.Z.); wyf1294005142@163.com (Y.-F.W.); 17881188229@163.com (Z.-W.S.); 2 Xinjiang Key Laboratory of Horse Breeding and Exercise Physiology, Urumqi 830052, China

**Keywords:** metagenome, conditioning training, Yili horse, microorganism

## Abstract

**Objectives**: The aim of this study was to investigate the effects of different stages of training on the intestinal microbial abundance of Yili horses. **Methods**: Ten Yili horses, all aged 2 years old and weighing 305 ± 20 kg, were selected and divided into a training group and an untrained group. The training group performed riding training 6 days a week, and the untrained group moved freely in the activity circle every day. Fecal samples were collected on days 30 and 60, and the intestinal microorganisms were detected and analyzed using metagenomics. **Results**: Compared with the 30-day untrained group, the relative abundances of Bacteroidetes were significantly increased in the 30-day training group (*p* < 0.01). Conversely, the abundances of *Clostridiaceae*, *Clostridium*, and *Ruminococcus* were significantly decreased (*p* < 0.01), whereas those of *Prevotella*, *Bacteroideaceae*, and *Bacteroidetes* were significantly increased (*p* < 0.05). Additionally, the relative abundances of Firmicutes and Actinomycetes were significantly decreased (*p* < 0.05). Compared with the 60-day untrained group, no significant differences in the phyla *Bacteriaceae* and *Bacteriae* of the 60-day training group (*p* > 0.05) were observed. In the linear discriminant analysis effect size analysis, seven significantly different bacteria were detected in the fecal flora of horses in the 30-day training group versus the untrained 30-day group, but only one significantly different bacterium was detected after 60 days. The Kyoto Encyclopedia of Genes and Genomes analysis showed that the differentially expressed genes were related to metabolism and the environmental information processing pathway, carbohydrate metabolism, and membrane transport pathways. **Conclusions**: Therefore, training seems to affect the diversity and composition of the gut microbiota of Yili horses, especially during the first 30 days of training.

## 1. Introduction

Yili horses are an excellent breed in China, suitable for both riding and pulling. They retain the advantages of being hardy and disease-resistant from Kazakh horses and other breeds, while integrating the superior structure and performance of international light horse breeds. This has resulted in a breed with professional value. Compared to other breeds, Yili horses have more advantages in nutritional feed, genetic breeding, and athletic performance. They are highly regarded due to their high production performance and research potential, with their athletic performance leading among domestic horses. The gut microbiota is crucial for the digestive system and overall health of horses. A deep understanding of the structure and function of bacterial communities is fundamental to exploring the mechanisms of horse gut health and disease. The microbiome consists mainly of bacteria, but also includes archaea, viruses, fungi, and protozoa. Microorganisms cover the surfaces of host mucous membranes, but most reside in the gastrointestinal tract. These microbial cell populations reach the highest density in the intestinal compartment and together form a complex microbial community known as the intestinal flora [1]. A growing number of studies from animals and humans have shown that the gut microbiota is an important factor influencing health and exercise performance in animals and humans [2]. The gut microbiota has direct and indirect effects on host physiology, including improving metabolic health and athletic performance [3]. Exercise significantly alters gut microbiota composition and diversity [4]. Physical activity is associated with the positive regulation of gut flora biodiversity, and gut microbes are involved in various interactions that affect health throughout the host’s life cycle. Physical exercise can regulate the gut microbiota through various mechanisms, such as by promoting neurotransmitter and hormone secretion, increasing intestinal transport [5].

Regular exercise induces physiological stressors that stimulate adaptive responses in the gastrointestinal tract and improve the integrity of the intestinal barrier. Cryan et al. [6] proposed a mechanism where exercise alters the gut microbiota: exercise-induced stress activates the hypothalamic–pituitary–adrenal axis, leading to the release of various hormones (e.g., adrenocorticotropic hormone, norepinephrine, and serotonin), which in turn affects the gastrointestinal environment and alters the gut microbiota. Many factors influence changes in the equine gut microbiota, such as age, sex, and mood, in addition to external factors, such as disease, transport, exercise, nutrition, management, probiotics, supplements, and medications. Horses are hindgut-fermenting herbivores that are dependent on billions of naturally occurring bacterial and protozoan fermentation products for the production of volatile fatty acids for energy production [7]. The intestinal tract of the horse contains several commensal bacteria, fungi, archaea, protozoa, and viruses [8], and is capable of hosting thousands of bacterial cells [9], with most bacteria being present in the colon and cecum [10].

High-quality sport horses need to be trained through specialized training to continuously improve sport performance in a specific direction, and horses that undertake regular specialized conditioning training show a series of physiological adaptive changes to achieve the optimal level for sport [11]. Metagenomics, initially conceptualized by Handelman, revolutionized microbial research by enabling the direct analysis of collective genomic data within microbial communities. This approach overcomes traditional limitations of microbial isolation and cultivation, particularly for unculturable species, thereby offering more accurate insights into in situ microbial diversity and ecological interactions. Furthermore, metagenomic techniques facilitate comprehensive investigations of metabolic networks and functional gene expression at a molecular resolution [12].

In this study, we analyzed the effects of different stages of conditioning training on the intestinal microorganisms of Yili horses based on metagenomics to provide a reference for improving the overall health and promoting the athletic performance of Yili horses, and enrich the theoretical system of genetic breeding and regulatory mechanisms of microorganisms related to exercise.

## 2. Materials and Methods

### 2.1. Experimental Animals and Groups

Ten free-grazing healthy Yili horses, all aged 2 years old and weighing 305 ± 20 kg, were selected from Zhaosu Racecourse in Yili, Xinjiang Province, China and they were randomly divided into a training group and an untrained group. The training group consisted of six horses and performed riding training 6 days a week. The untrained group consisted of four horses and were free to move around the activity pen every day. All horses were fed the same diet and kept in the same rearing environment under unified management after the trial began.

### 2.2. Sample Collection

#### 2.2.1. Fecal Samples

Fecal samples were collected from the ten test horses using the rectal collection method at rest on days 30 and 60 of the trial; it consists of six trained-group samples and four untrained-group samples at each time point, all samples were collected at the same time of day, and the collected samples were snap-frozen and stored in liquid nitrogen. The samples collected on day 30 were recorded as “QY” for the trained group and “QB” for the untrained group; the samples taken on day 60 were recorded as “HY” for the trained group and “HB” for the untrained group.

#### 2.2.2. Sample Results from the 2000 m Test Race

Horses in the training group performed a test race on a 2000 m circular sand track on days 0, 30, and 60 of the trial, and the results were recorded.

### 2.3. Sample Quality Control

The genomic DNA of the sample was extracted using an extraction method consistent with the sample type (Tengen Magnetic Bead Kit, Thermo Fisher, Dreieich, Germany). The purity and integrity of the DNA was analyzed using 1% agarose gel electrophoresis. After DNA quantification, an appropriate amount of sample was collected in a centrifuge tube, the sample was diluted to the required concentration, and DNA purity was confirmed by measuring the A260/A280 ratio, which ranged between 1.8 and 2.0.

### 2.4. Library Construction, Quality Control, and Sequencing

The genomic DNA was randomly fragmented into short pieces, and sequencing libraries were constructed. The fragments underwent end repair, A-tailing, and adapter ligation using Illumina adapters. Following adapter ligation, the fragments were PCR-amplified, size-selected, and purified. Library quality was assessed by Qubit quantification, real-time PCR for concentration measurement, and bioanalyzer analysis for size distribution. Based on the determined library concentration and desired data volume, the libraries were pooled and subjected to sequencing on Illumina platforms.

### 2.5. Bioinformatics Analysis Pipeline

Raw Illumina HiSeq data were preprocessed using Readfq to generate clean reads. Host contamination was removed via Bowtie2 alignment against host genomes. Metagenome assembly was performed per sample, followed by gene prediction using MetaGeneMark. A non-redundant gene catalog was constructed by dereplicating predicted genes across samples. Gene abundances were quantified by mapping reads from each sample to the catalog. Taxonomic annotation was performed against the MicroNR database, while functional annotation utilized KEGG, eggNOG, and CAZy. Community and functional profiles were analyzed via PCA, NMDS, clustering, and ANOSIM. For grouped samples, differential abundance (Metastat, LEfSe) and metabolic pathway comparisons were conducted.

### 2.6. Statistical Analysis

Statistical comparisons of dominant microbial taxa (top 10 most abundant at phylum, family, and genus taxonomic levels) were performed using independent two-sample t-tests implemented in SPSS version 26.0, adopting a conservative significance threshold of *p* < 0.01. To further characterize these microbial community variations, we conducted effect size estimation through linear discriminant analysis (LDA) as part of the LEfSe analytical pipeline. A Spearman correlation analysis of microbial communities was performed using the “Hmisc” package, with the false discovery rate (FDR) correction for *p*-values implemented via the “fdrtool” package, and with *p* < 0.05 indicating significant differences, and *p* > 0.05 indicating nonsignificant differences.

## 3. Results

### 3.1. Test Match Results

As shown in Table 1, the Yili horses in the training group showed a significant improvement in the 30th and 60th day test race performance compared with the previous stage (*p* < 0.05), and the test race performance on the 60th day showed a highly significant improvement compared with day 0 (*p* < 0.01).

### 3.2. Statistical Results of the Macrogenome Sequencing Data

As shown in Table 2, the valid sequences of the four groups of data, Q20 and Q30, were above 98% and 93%, respectively, indicating that the sequencing data had high reliability. The open reading frame predictions for each sample and mixed assembly were de-redundant to obtain a nonredundant initial gene catalog of 6,063,836 for subsequent abundance analysis.

### 3.3. Analysis of Differences in the Number of Genes

**Figure 1:**
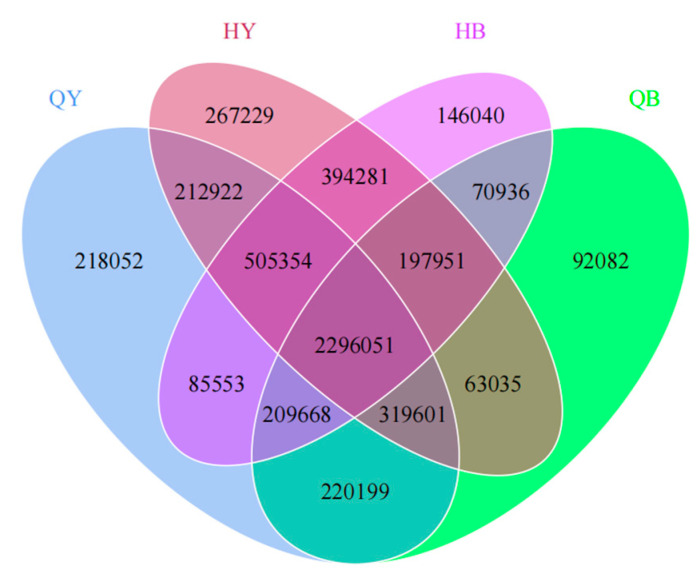
Wayne diagram of the gene number.

As shown in Figure 1, the number of genes shared between the four groups was 2,296,051, and the number of genes unique to the trained group (30 and 60 days for the training group) was higher than that of the untrained group. The number of genes unique to the 30-day training group was 218,052, and the number of genes unique to the 30-day untrained group was 92,082. The number of genes unique to the 60-day training group was 267,229, and the number of genes unique to the untrained 60-day group was 146,040. The greatest change in the number of genes was observed in the 60-day training group in this experiment, followed by the 30-day training group.

### 3.4. Analysis of the Relative Abundance of Different Levels of Fecal Flora in Yili Horses

**Figure 2:**
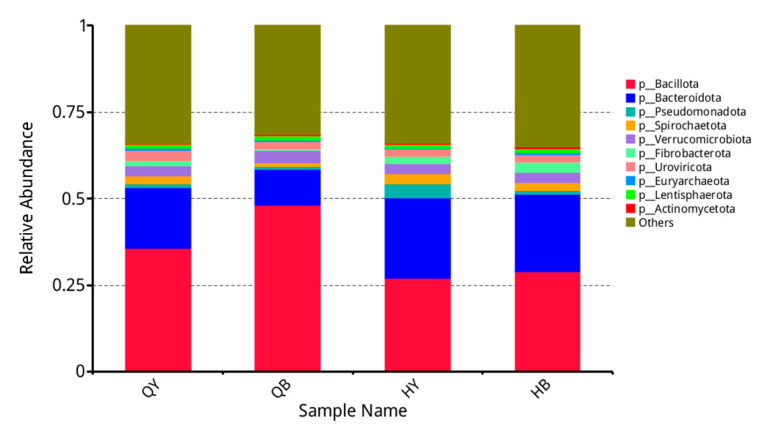
Analysis of the relative abundance of fecal flora at the phylum level.

As shown in Figure 2, the top ten species in terms of relative abundance at the phylum level were *Bacillota*, *Bacteroidota*, *Pseudomonadota*, *Spirochaetota*, *Verrucomicrobiota*, *Fibrobacterota*, *Uroviricota*, *Euryarchaeota*, *Lentisphaerota*, and *Actinomycetota*. Compared with the 30-day untrained group, the 30-day training group showed a highly significant increase in the relative abundance of *Bacteroidota* (*p* < 0.01), a significant decrease in the relative abundance of *Bacillota* and *Actinomycetota* (*p* < 0.05), and no significant difference in the rest of the phyla (*p* > 0.05).

**Figure 3:**
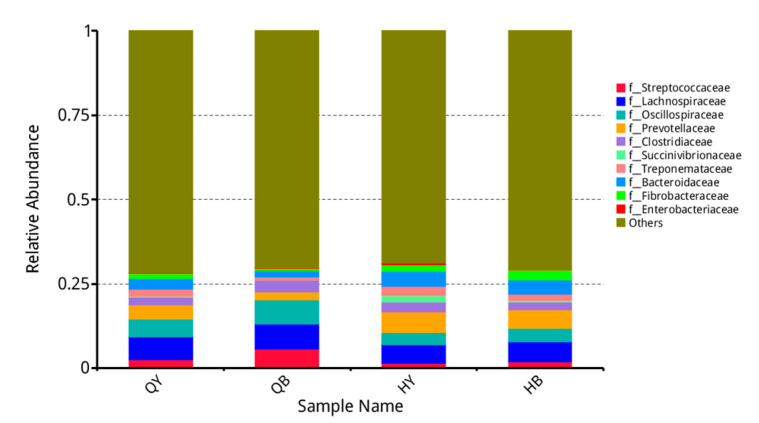
Analysis of the relative abundance of fecal flora at the family level.

As shown in Figure 3, at the family level, the top ten organisms in terms of relative abundance were *Streptococcaceae*, *Trichoderma*, *Treponema*, *Prevotella*, *Clostridiaceae*, *Vibrio succinicola*, *dense Spirochaetes*, *Bacteroidota*, *Fibrobacteria*, and *Enterobacteriaceae*. The relative abundance of *Clostridiaceae* was extremely significantly lower (*p* < 0.01) in the 30-day training group than in the untrained group (*p* < 0.05), and the relative abundance of *Prevotella* and *Synechococcaceae* was significantly higher (*p* < 0.05), whereas the other families were not significantly different (*p* > 0.05).

**Figure 4:**
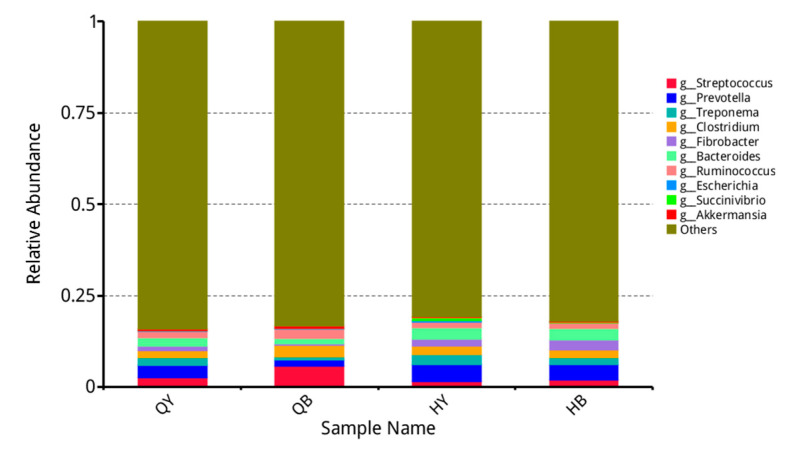
Analysis of the relative abundance of horizontal fecal flora in the genus Ilioma.

As shown in Figure 4, at the genus level, the top ten organisms in terms of relative abundance were *Streptococcus*, *Prevotella*, *Treponema*, *Clostridium*, *Fibrobacter*, *Bacteroides*, *Ruminococcus*, *Escherichia*, *Succinivibrio*, and *Akkermansia.* The relative abundance of *Clostridium* and *Ruminococcus* was significantly lower (*p* < 0.01) in the 30-day training group than in the untrained 30-day group, and the relative abundance of *Prevotella* and *Bacteroides* was significantly higher (*p* < 0.05), whereas the remaining organisms did not show any significant differences (*p* > 0.05).

### 3.5. Principal Component Analysis of Intestinal Microbial Species Abundance in Yili Horses

**Figure 5:**
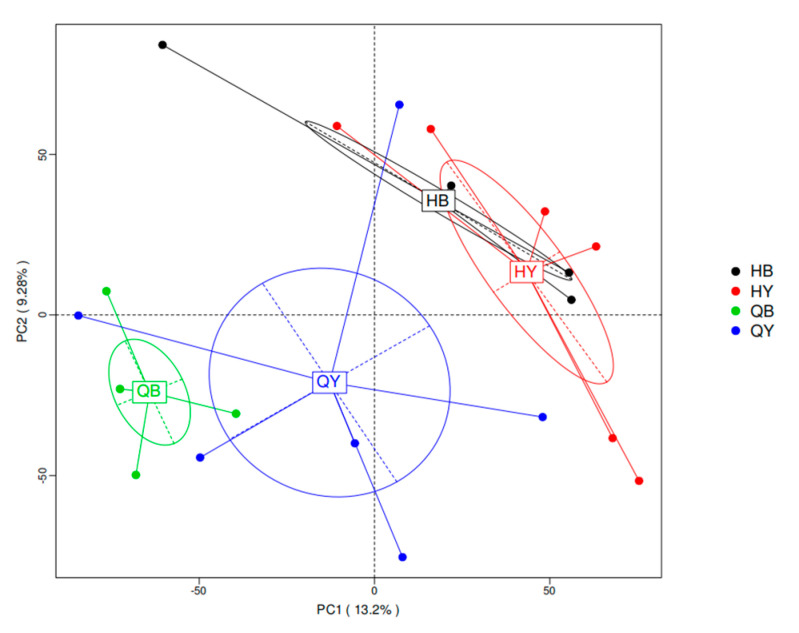
Results of species-based PCA. Note: Horizontal coordinates indicate the first principal component, vertical coordinates indicate the second principal component, and percentages indicate the contribution of the principal component to the sample variance. Each point in the graph indicates a sample, and samples from the same group are indicated using the same color.

As shown in Figure 5, the PCA of species abundance at the species level showed that both the 30-day trained and untrained groups clustered alone and significantly away from each other, indicating that the composition of gut microbes between the two groups differed significantly at the species level. In contrast, the 60-day trained and untrained groups were arranged in a more dispersed manner, suggesting that there were no significant differences in the composition of gut microbes between the two groups at the species level at 60 days.

### 3.6. Linear Discriminant Analysis Effect Size Analysis of Species Differing Between Groups

**Figure 6:**
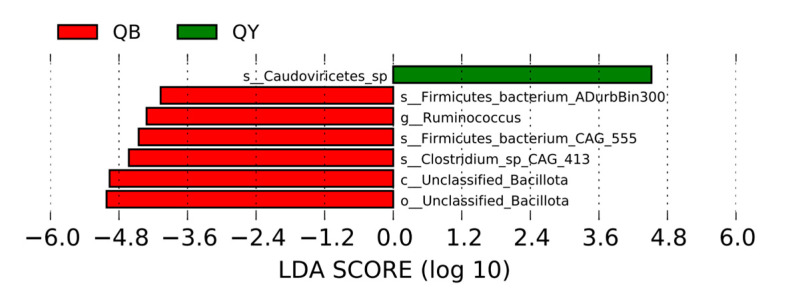
LEFSe analysis histogram of the fecal flora of Yili horses in the 30-day training group and the 30-day untrained group.

As shown in Figure 6, seven significantly different bacteria were detected in the fecal flora of the horses in the 30-day training group versus the 30-day untrained group. One species was significantly upregulated in the fecal flora of Yili horses in the 30-day training group, a tailed phage (Caudoviricetes); six species were significantly upregulated in the fecal flora of Yili horses in the untrained 30-day group (Firmicutes_bacterium_ADurbBin300, Firmicutes_bacterium_CAG_555, Clostridium_sp_CAG_413, unclassified bacteria of Mycetae, and unclassified bacteria of Bacteriales).

**Figure 7:**
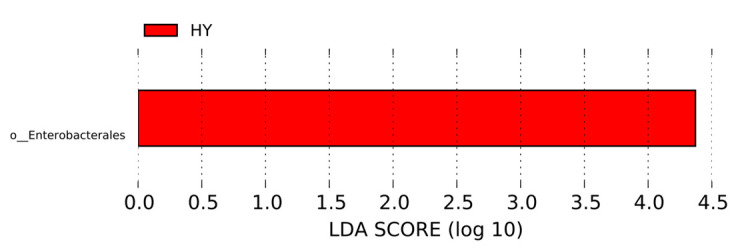
LEFSe analysis of fecal flora of Yili horses in the 60-day training group and the 60-day untrained group.

As shown in Figure 7, only one type of bacteria in the fecal flora of Yili horses differed between the 60-day training group and the 60-day untrained group: Enterobacteria were significantly enriched in the fecal flora of Yili horses in the 60-day training group.

### 3.7. Notes on the Intestinal Microbiome Function of Yili Horses

#### 3.7.1. Annotated Gene Number Statistics

**Figure 8:**
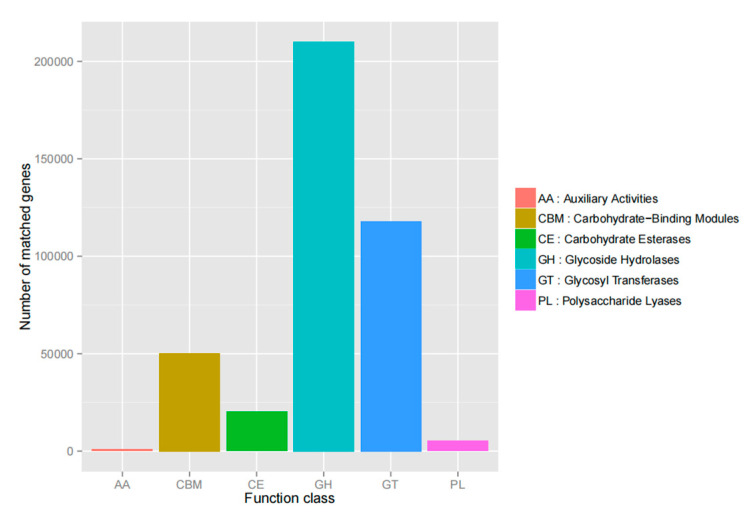
Statistical map of the number of annotated genes.

Using the CAZy database as a reference, the number of annotated genes in each database was plotted. As shown in Figure 8, the glycoside hydrolase class had the highest number of genes, followed by glycosyltransferases (GT), carbohydrate binding modules, and carbohydrate esterases, and the least number of polymers, lysosomal lyases, and co-activator enzyme lines (AA).

#### 3.7.2. Functional Analysis

**Figure 9:**
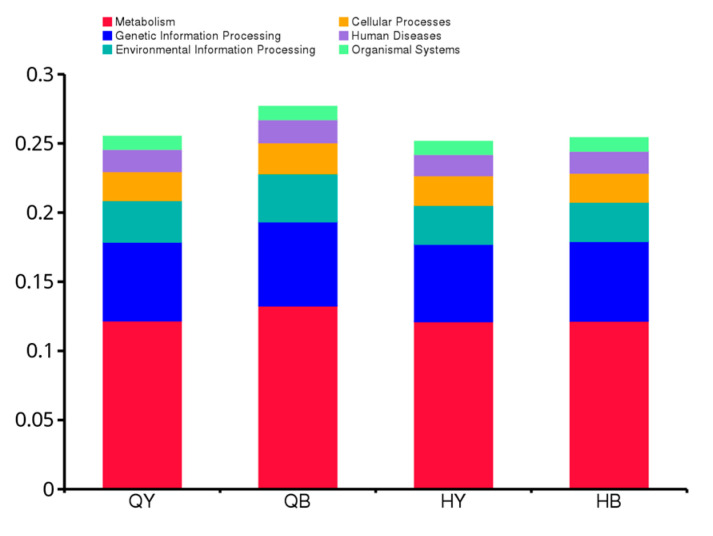
Histogram of the relative abundance of functional annotations at level 1.

**Figure 10:**
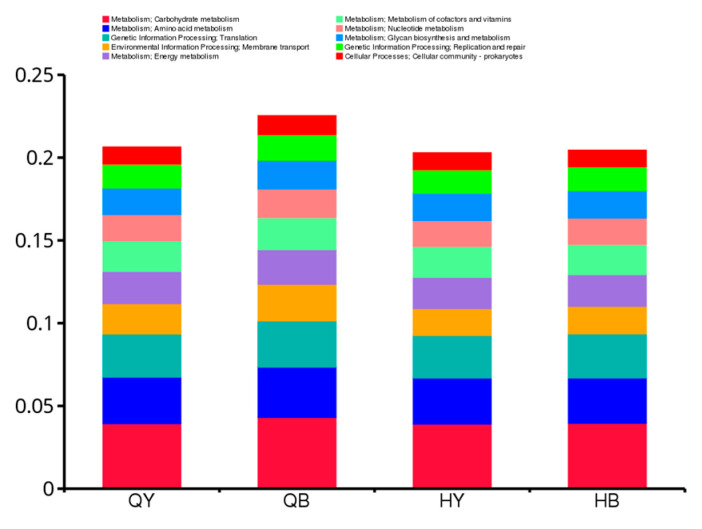
Histogram of the relative abundance of functional annotations at level 2.

Based on the annotation results from the KEGG database, as shown in Figure 9, the highest number of genes annotated at level 1 were related to metabolism, and the lowest number of genes were related to organismal systems. As can be seen in Figure 10, six of the top ten results in terms of relative abundance at level 2 were related to metabolic pathways, with the highest numbers of annotated genes, in descending order, being related to carbohydrate metabolism, amino acid metabolism, translation, membrane transport, energy metabolism, cofactor and vitamin metabolism, nucleotide metabolism, polysaccharide biosynthesis and metabolism, replication, repair, and cellular communities of prokaryotes. At level 1, significant differences in metabolism and environmental information processing pathways were noted between the trained 30-day group and the untrained 30-day group (*p* < 0.05), and at level 2, significant differences were observed in carbohydrate metabolism and membrane transport pathways between the trained 30-day group and untrained 30-day group (*p* < 0.05). The other groups showed no significant differences.

#### 3.7.3. Functional Relative Abundance Cluster Analysis

**Figure 11:**
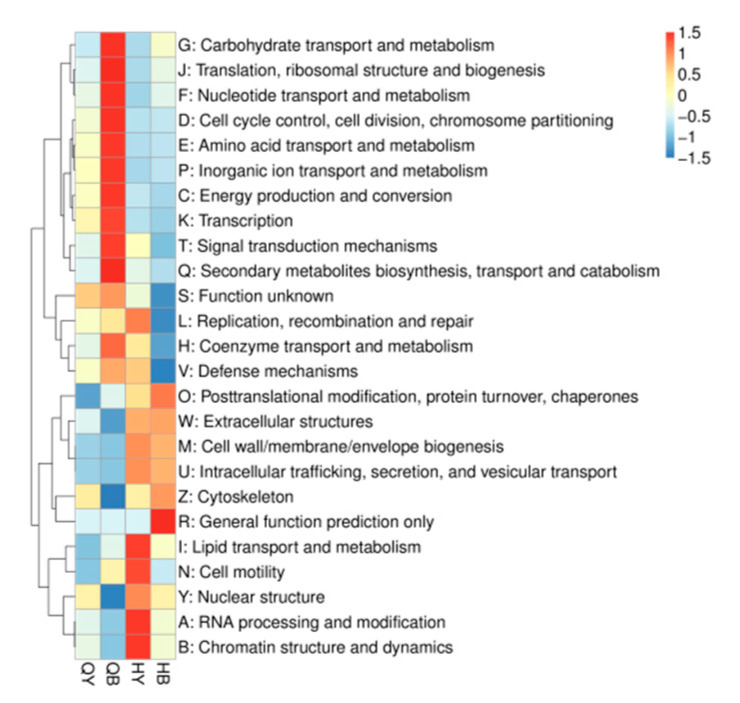
Functional abundance clustering heatmap.

Based on EggNOG data analysis, the top 25 functions in terms of abundance and their abundance information in each sample were selected to draw heatmaps (Figure 11) and clustered at the level of functional differences. In addition to unknown functions, the highest number of annotated genes were found in replication, recombination, and repair, followed by carbohydrate transport and metabolism, translation, ribosome structure and biogenesis, and transcription.

## 4. Discussion

The gut microbiota and its associated metabolites may function locally in the gut or accumulate in different body fluids, thereby affecting the host’s athletic performance during competition [13,14]. The gut of the horse contains a rich and complex microbial community, which plays an important role in the physiology, metabolism, nutrition, and immune function of the horse [15]. The intestinal flora is regulated by immutable factors (e.g., sex, genetics, age, and race) and modifiable factors (e.g., host health, activity, diet, and antibiotics). Exercise can determine changes in gut microbial composition and plays an active role in energy homeostasis and regulation [16]. Exercise training is one of the factors that affect the gut flora. Evans et al. [17] showed that exercise training affected the composition of microbial communities in the gastrointestinal tract of mice while Clarke et al. [18] showed that the diversity of gut flora was significantly higher in athletes than in controls matched for body size, age, and sex. In the current study, the results showed that the number of genes was higher in the 60-day training group than in the 30-day training group, suggesting that long-term regular training affects the abundance of the intestinal flora [19]. The results showed that at 30 days of training, there were more significantly different strains of bacteria in the trained group than in the untrained group, whereas at 60 days of training, there were no significantly different strains of bacteria in the trained group compared with the untrained group, which was in agreement with the results of the study by Janabi et al. [20]. Pre-exercise training affects the intestinal microbiota of the horse, but these changes are reversed in the later stages of training, showing that the gut microbiota develops temporal and exercise-induced adaptive responses, ultimately reaching a stable equilibrium without significant subsequent variations.

The majority of the healthy adult microbiota is controlled by *Bacillota* and *Bacteroidota*, which together constitute the majority of bacterial taxa in microbiology. Other taxa include Ascomycetes, Actinobacteria, Clostridia, warty *Micromycetes*, methanogenic archaea, eukaryotes (protists and fungi), and other colonizers. There are a wide variety of bacterial species in the living world, and of all the known bacterial classes, only two phyla dominate the mammalian gut: *Bacteroidota* (16.3%) and *Bacillota* (65.7%) [21]. *Bacteroidota* is a major member of the human and animal intestinal microbial community [22], which, together with Thickettsia, forms a unique symbiotic relationship, with each occupying a different position in the intestinal microbial ecosystem. Together, they not only participate in the digestive process but also influence the immune system and maintain intestinal homeostasis through the production of specific metabolites. The dominant phyla in the equine intestine are mainly the thick-walled and anamorphic bacteria, where *Bacillota* are the optimal phyla in the equine intestinal tract, with their proportion of relative abundance in the intestine ranging from 40% to 90% [23]. Exercise performance is strongly correlated with gut flora, and athletes have a high abundance of gut microorganisms such as *Veillonella*, *Bacteroides*, and *Prevotella*. [24]. Liu et al. [25] found changes in the Firmicutes and Proteobacteria after exercise training in rats. Morita et al. [26] studied a group of healthy elderly women divided into trunk muscle training and whole-body aerobic exercise training, and found that the elderly women who underwent aerobic exercise training showed a significant increase in the species of Bacteroidetes in their intestinal tracts. In the current study, the results showed that the dominant organisms in the feces of horses in the 30-day training group, 30-day untrained group, 60-day training group, and 60-day untrained group were *Bacillota*, *Bacteroidota*, *Pseudomonas*, *Spirochaetes*, and *Micrococcus verrucosus*. The dominant families were *Streptococcaceae*, *Trichoderma*, *Treponema*, *Prevotella*, and *Clostridia*. The dominant genera were *Streptococcus*, *Prevotella*, *Mycosphaerella*, *Clostridium*, and *Fibrobacterium*. A highly significant increase in the relative abundance of *Bacteroidota* and a significant decrease in the relative abundance of *Bacillota* in the intestinal flora of Yili horses in the 30-day training group compared with the 30-day untrained group are in agreement with the findings of Emmanuel et al. [27], who found that, after 6 weeks of exercise training, the intestinal flora of mice changed, with an increase in the abundance of *Bacteroidota* and a decrease in the abundance of *Bacillota*. The type and intensity of exercise affects the composition of the equine intestinal flora, and strenuous exercise may increase the abundance of dense Helicobacter spp. and *Clostridium* in horses [28]. Plancade [29] and Janabi et al. [20] found that training induced an increase in the abundance of intestinal dense Helicobacter in endurance horses, and the results of the present experiment showed that, in comparison with the untrained 30-day group, the relative abundance of dense *Spirochete* spp. in the intestinal flora of Yili horses in the 30-day training group was increased, but the difference was nonsignificant while the relative abundance of *Clostridium* was significantly decreased, which was not in complete agreement with the results of the previous studies and may be due to the factors of the intensity of the exercise and the individual differences of horses between this trial and the previous studies. Scheiman et al. [13] found that the microbiome of athletes contained a diverse composition of microorganisms, characterized by an elevated abundance of microporaceae, mycobacteriaceae, prevotella, methanobacterium, and ackermannia. The abundance of taxa involved in energy and carbohydrate metabolism, such as *Prevotella* and *Methanobacterium smithi*, has been found to be significantly higher in professional cyclists than in amateur cyclists and correlates with training frequency [24]. *Prevotella* is positively correlated with the metabolic pathway of branched-chain amino acids in the microbiome, which are essential amino acids that promote muscle protein synthesis for postexercise recovery. Hollister et al. [30] found that a small amount of Proteobacteria and a large amount of *Bacteroidota*, *Prevotella*, and *Luminococcus* spp. were beneficial to health, which is similar to the results of this experiment, where a highly significant increase in the relative abundance of Proteobacteria was detected in the intestinal flora of the Yili horses in the 30-day training group, as well as a significant increase in the relative abundance of *Bacteroidota* and *Prevotella* spp., which suggests that training increases the abundance of *Bacteroidota* and *Prevotella* in the intestinal flora. These organisms promote energy metabolism, and therefore improve the health level of Yili horses and promote their performance. The effect of exercise on the microbiome is closely related to the intensity and duration of exercise, and training may not only enhance this effect but may also produce entirely new effects. Changes in the diversity and composition of the gut microbiota can reduce inflammation, while altering hundreds of beneficial metabolites, such as short-chain fatty acids and secondary bile acids, which can promote exercise performance.

The functional gene annotation results based on the KEGG database revealed significant differences in metabolic and environmental information processing pathways at level 1 and carbohydrate metabolism and membrane transport pathways at level 2 between the trained and untrained 30-day groups. Energy production and utilization is vital during exercise, and carbohydrates play a key role in this process. As a primary source of energy, carbohydrates support athletic performance by providing a consistent and steady energy output. Carbohydrates also serve to conserve protein and fat during exercise, thus reducing muscle damage and recovery time after exercise. The functional relative abundance results indicated that the gut microbial functions mainly included carbohydrate and amino acid metabolism, translation, membrane transport, and energy metabolism. Training can influence the composition and function of the gut microbial community of Yili horses, providing a theoretical basis for understanding the gut microbial community of Yili horses.

## 5. Conclusions

In this study, we found that conditioning training affected the gut microbiota of Yili horses, particularly during the pretraining phase (30 days), and that these changes stabilized during the post-training phase (60 days). The diversity and composition of the intestinal flora were richer in the trained group than in the untrained group, with an increase in the relative abundance of Bacteroidetes and a decrease in the relative abundance of Bacillota in the intestinal tract.

The present study elucidates potential mechanisms by which exercise may enhance performance and benefit health in Yili horses. Future investigations on training-induced alterations in fecal microbiota should incorporate critical training variables (frequency, intensity, duration, and volume) to establish a comprehensive theoretical framework of exercise–microbiome interactions. Such mechanistic studies will be essential for fully characterizing the relationship between training regimens and gut microbial ecology responses in this equine breed.

## Figures and Tables

**Table 1:** 2000 m trotter score.

Group	Trained Group	Untrained Group
Results of the 30th test event (s)	302.33 ± 21.30 B	366.04 ± 25.46 A
Results of the 60th test event (s)	290.64 ± 15.59 B	357.93 ± 10.22 A

Note: Different uppercase letters denote highly significant differences at *p* < 0.01. Shared superscript letters or absence of letters indicate nonsignificant differences (*p* > 0.05).

**Table 2:** Sequencing statistics.

Sample Name	Raw Reads	Clean Reads	Q20/%	Q30/%	GC/%	Effective Rate/%
Q.72Y	6653.59	6554.32	98.47	94.97	47.27	98.508
Q.80Y	6729.13	6618.38	98.21	94.2	43.64	98.354
Q.88Y	6645.57	6525.99	98.31	94.55	47.8	98.201
Q.93Y	6492.36	6393.68	98.42	94.81	45.63	98.48
Q.104Y	6838.43	6733.09	98.31	94.45	42.56	98.46
Q.105Y	6929.09	6783.72	98.05	93.83	38.81	97.902
Q.73B	5963.66	5856.69	98.22	94.28	45.98	98.206
Q.86B	5859.63	5768.79	98.41	94.78	45.19	98.45
Q.91B	5925.94	5809.46	98.08	93.88	46.05	98.034
Q.97B	6220.65	6125.11	98.35	94.61	46.4	98.464
H.72Y	6696.04	6577.68	98.22	94.26	45.86	98.232
H.80Y	6825.94	6731.50	98.41	94.73	42.8	98.616
H.88Y	6839.76	6737.69	98.42	94.79	44.2	98.508
H.93Y	6459.27	6365.68	98.41	94.78	43.8	98.551
H.104Y	6294.44	6195.70	98.4	94.75	44.31	98.431
H.105Y	6178.19	6054.10	98.19	94.18	43.05	97.991
H.73B	6595.90	6470.27	98.35	94.62	43.59	98.095
H.86B	6634.70	6540.49	98.39	94.69	43.72	98.58
H.91B	6458.46	6351.98	98.23	94.27	43.47	98.351
H.97B	6687.72	6567.63	98.31	94.54	46.37	98.204

## Data Availability

The original contributions presented in this study are included in the article. Further inquiries can be directed to the corresponding author.
